# Liver and Plasma Fatty Acid Characterization in Cultured Brown Trout at Distinct Reproductive Stages

**DOI:** 10.3390/biology12111434

**Published:** 2023-11-15

**Authors:** Tânia Vieira Madureira, Diana Santos, Tiago Simões, Marco F. L. Lemos, Eduardo Rocha

**Affiliations:** 1 Team of Animal Morphology and Toxicology, Interdisciplinary Centre of Marine and Environmental Research (CIIMAR/CIMAR), University of Porto (U. Porto), Terminal de Cruzeiros do Porto de Leixões, Av. General Norton de Matos s/n, 4450-208 Matosinhos, Portugal; dianasantiagosantos@gmail.com (D.S.); erocha@icbas.up.pt (E.R.); 2 Laboratory of Histology and Embryology, Department of Microscopy, ICBAS—School of Medicine and Biomedical Sciences, University of Porto (U. Porto), Rua Jorge Viterbo Ferreira 228, 4050-313 Porto, Portugal; 3 MARE—Marine and Environmental Sciences Centre & ARNET—Aquatic Research Network Associated Laboratory, ESTM, Polytechnic of Leiria, 2520-641 Peniche, Portugal; tiago.simoes@ipleiria.pt

**Keywords:** brown trout, fatty acids, liver, plasma, reproductive stages

## Abstract

**Simple Summary:**

Lipids, namely fatty acids, are crucial energy sources in fish development, reproduction, and migration, among other processes. In this study, we are interested in identifying the most relevant fatty acids in the liver and plasma throughout the reproductive cycle of farmed brown trout in both sexes. Four reproductive stages along the cycle were selected: spawning capable (December), regressing (March), regenerating (July), and developing (November) stages. The most abundant fatty acids were palmitic acid (16:0), stearic acid (18:0), oleic acid (18:1 n-9), arachidonic acid (20:4 n-6), eicosapentaenoic acid (EPA, 20:5 n-3), and docosahexaenoic acid (DHA, 22:6 n-3). Sex differences were found for some fatty acids, but seasonal changes stood out more. In the liver, polyunsaturated fatty acid (PUFA) levels were significantly higher in December than in March and July for both males and females. Changes between the reproductive stages in male and female plasma were mostly related to SFA and PUFA, and PUFA and ∑n-3 PUFA, respectively. This evidences that PUFAs are crucial for reproduction in both sexes. Further, in female plasma samples, the most representative fatty acids distinguished the four reproductive stages, which indicates that those fatty acids should be further investigated as biomarkers of female maturity statuses.

**Abstract:**

Fatty acids are energy sources, and their profiles are used as biomarkers of metabolic status and physiological changes in fish. Within this context, the main aim of this study was to identify the fatty acids that best discriminate the reproductive status of male and female farmed brown trout. The fatty acid composition in liver and plasma samples from the adults of both sexes was monitored along four distinct reproductive stages, namely the spawning capable (December), regressing (March), regenerating (July), and developing (November) stages. Irrespective of the sex and stage, the most representative fatty acids were palmitic acid (16:0), stearic acid (18:0), oleic acid (18:1 n-9), arachidonic acid (20:4 n-6), eicosapentaenoic acid (EPA, 20:5 n-3), and docosahexaenoic acid (DHA, 22:6 n-3). There were no significant sex differences in fatty acid classes in the liver and plasma. Despite this, there were several changes in individual fatty acid levels between the sexes. In the liver, both males and females showed high monounsaturated fatty acid and low polyunsaturated fatty acid (PUFA) levels during the regressing and regenerating stages. At spawning capable and developing stages, a reverse profile was noted. The plasma profiles were mainly influenced by changes in saturated fatty acids and PUFAs in males and by PUFA in females. Based on the most representative fatty acids, four patterns were established for female plasma samples, one for each reproductive stage. This scenario suggests that female plasma samples are promising for the discrimination of gonadal reproductive status, and this potential can be further explored in aquaculture and environmental monitoring studies.

## 1. Introduction

Fatty acid analyses have been widely used in fish research for distinct purposes. Some studies focus on fatty acid composition to discriminate freshwater versus seawater fish [1], wild versus cultured specimens [2], or colder versus warmer fish habitat conditions [3]. Under these conditions, some patterns stand out. Data support that freshwater fish contain higher levels of n-6 polyunsaturated fatty acids (PUFAs) and lower levels of n-3 PUFAs than seawater specimens, resulting in lower n-3/n-6 ratios [4,5]. From another perspective, wild brown trout showed higher muscle PUFA and lower monounsaturated fatty acid (MUFA) contents than farmed fish [2]. The n-3 PUFA levels were higher and n-6 PUFA were lower in wild individuals compared to farmed ones. In colder habitats, fish had higher levels of n-3 PUFA, which is more appealing for human consumption [6].

Apart from these mentioned examples, many other parameters can highly influence fatty acid composition changes in different fish tissues, but one of the most relevant is the reproductive status of fish [7]. One key reason is that fish spawning capacity is highly influenced by PUFA contents [8]. This point has direct progeny consequences since fish egg fatty acid compositions compromise fertility rates, hatching, and, larva growth [9]. Notably, many studies on fish fatty acid profiles did not discriminate the sex or only used female fish, presumably because females are more likely to change. Even though the fatty acid composition may be sex-independent, there may be an interference due to sex in the profiles of fish, as demonstrated in macrostigma trout (*Salmo trutta macrostigma*) [10].

Most studies focus on quantifying fatty acids in the muscle because the main goal is to assess nutritional fish impacts on human health, namely in terms of n-3 PUFA intake. By contrast, the fatty acid dynamics between liver and plasma levels are unexplored. The liver has a crucial role in fish lipid metabolism, and its components are exported into the bloodstream and coordinate gonad maturation. When it comes to fatty acid composition, plasma/serum is the most stable blood fraction during storage, as reviewed by Metherel and Stark (2016) [11]. The interconnection between the liver and gonads was demonstrated with the example of female triploid rainbow trout (*Oncorhynchus mykiss*), in which the absence of ovaries interfered with the process of the hepatic synthesis of fatty acids, particularly PUFAs and MUFAs [12]. Furthermore, the same species from Derbent Dam Lake had more fatty acids in the liver than in the muscle [13]. Interestingly, in macrostigma trout, the fatty acid contents in the liver expressed differences between males and females that were more striking than in the muscle [10]. Up until now, seasonal changes in liver fatty acid contents have been found, for example, in the European chub (*Squalius cephalus*) [14], pikeperch (*Sander lucioperca*) [15], catfish (*Silurus triostegus*) [16], and rainbow trout [13]. Female rainbow trout liver evidenced a high accumulation of 22:6n-3 (docosahexaenoic acid, DHA) during vitellogenesis [7], which has been identified as a crucial fatty acid for egg quality and larvae viability in fish [17].

Within the explained context of potential seasonal and inter-sexual differences, this study aims to evaluate the total plasma and liver fatty acid contents in four reproductive phases of cultured brown trout adult specimens from both sexes. This work expands on a previous one that proposed the analysis of (*sic.*) “the fitness and gonadal maturation” of these animals using histological, biometric, biochemical, and hormonal parameters [18]. Differently, the present study comprises the first detailed fatty acid profile and temporal follow-up of fatty acid dynamics in both types of biological matrices, serving the goal of identifying the classes and individual fatty acids that can be used as biomonitoring tools to discriminate the reproductive status of male and female brown trout. The data may also be viewed as a baseline of normal values against which stressor impacts may be compared.

## 2. Materials and Methods

### 2.1. Animals

Brown trout (3–4 years old) of both sexes were reared and maintained in a state-controlled aquaculture facility (Torno Station, Amarante, Portugal), according to the detailed description in a previous study [18]. Fish were kept under a natural photoperiod and fed with commercial trout pellets (ICNF7MM, AQUASOJA) throughout the study time. A sampling of six fish per sex that were of the same age cohort was performed along each of the four reproductive stages: December (2017, 3-year-old cohort), spawning capable; March (2018, age cohort 3+), regressing; July (2018, age cohort 3+), regenerating; and November (2018, new 3-year-old cohort), developing. These stages were established according to reported criteria [19] and as previously used in brown trout [18]. The sampling was executed on site, and animal handling followed the Portuguese Decree-Law No. 113/2013, translating EU Directive No. 2010/63 with respect to animal protection for scientific purposes.

Mean total fish weights (standard deviation) in the four reproductive stages were as follows: December, 783.3 ± 115.5 g (males) and 674.3 ± 174.6 g (females); March, 1290.0 ± 362.4 g (males) and 1343.0 ± 447.1 g (females); July, 1596.0 ± 473.8 g (males) and 1347.0 ± 597.9 g (females); and November, 863.0 ± 283.5 g (males) and 780.0 ± 130.8 g (females). The mean fish lengths were as follows: December, 41.0 ± 1.5 cm (males) and 38.9 ± 3.8 cm (females); March, 45.3 ± 4.3 cm (males) and 47.2 ± 4.9 cm (females); July, 50.8 ± 4.4 cm (males) and 46.8 ± 6.2 cm (females); and November, 41.4 ± 4.3 cm (males) and 41.0 ± 1.9 cm (females). The details of the measurement and characterization procedures of the fish along the distinct stages were previously published [18].

### 2.2. Sampling Procedures

Euthanasia was carried out individually for each fish in a single tank, using an overdose solution of ethylene glycol monophenyl ether (2 to 3 mL/L) (Merck, Darmstadt, Germany) in water taken from aquaculture systems. Then, fish were weighed and measured, and whole blood was collected into lithium-heparin-coated tubes (Vacuette LH Lithium Heparin, Greiner Bio-One, Kremsmünster, Austria). Plasma (300 µL) was obtained after centrifugation (2000× *g* for 20 min), snap-frozen in liquid nitrogen, and stored at −80 °C. A liver fragment (300 mg) was sampled, snap-frozen in liquid nitrogen, and stored at −80 °C.

### 2.3. Plasma and Liver Fatty Acids Analyses

Plasma fatty acid analyses followed a previously implemented methodology for plasma samples from juvenile brown trout [20], which was first developed by Silva et al. (2017) [21]. For liver fatty acid analyses, about 75 mg of tissue was used for the extraction, following the same methodology implemented for plasma samples with minor modifications.

Firstly, the alkaline hydrolysis of more complex fatty acid esters was performed by adding 500 µL of 2 M KOH (diluted in 67% ethanol; *v*/*v*) to each sample. Samples were then kept at 80 °C for 1 h, cooled to room temperature, diluted 1:1 with water, and acidified to pH = 1 using HCl (5N). Hexane (500 μL) was added to isolate the FAs in an organic phase. Samples were stirred and centrifuged for 5 min at 1500× *g*, and the upper organic phase was collected.

After the alkaline hydrolysis of FA fractions isolated from each sample, 3 mL of acetyl chloride–methanol solution (1:20 *v*/*v*) and the internal standard (nonadecanoic acid—10 mg/mL) were added before FA derivatization. The samples were kept at 80 °C for 1 h and allowed to cool down to room temperature. Afterwards, 1 mL of Mili-Q water and 1 mL of n-heptane were added. Samples were stirred for 1 min, and the organic layer was collected to clean vials before analysis. The operating conditions were set as described in [21,22] with a few modifications. A Finnigan Ultra Trace gas chromatograph and a flame ionization detector (FID) equipped with a Thermo TR-FAME capillary column (60 m × 0.25 mm ID, 0.25 μm film thickness), coupled to an autosampler AS 3000 from Thermo Electron Corporation (Waltham, MA, USA), were used to analyze the FA methyl esters. The injector (operating in splitless mode) and detector temperatures were set at 250 and 280 °C, respectively. The column’s temperature was initially set at 100 °C for 1 min and then raised at 10 °C/min to 160 °C and held for 10 min, followed by an increase at 4 °C/min to 235 °C and maintained for 10 min. Helium was used as carrier gas at a flow rate of 1.5 mL/min. Air and hydrogen were supplied to the FID detector at flow rates of 350 and 35 mL/min, respectively. FA methyl ester mixes (PUFA No1 from marine source, PUFA No 3 from menhaden oil, and a 37-component FAME mix) were used as external standards (Supelco, Bellefonte, PA, USA). The theoretical correction factor (FCT) for FID detectors was applied in FA quantification [23].

The amount of each fatty acid or class in the liver and plasma samples is given as a percentage. The following fatty acid profiles were quantified in both plasma and liver samples: saturated fatty acids—SFA (12:0, 13:0, 14:0, 15:0, 16:0, 17:0, 18:0, 20:0, 22:0 and 24:0); monounsaturated fatty acids—MUFA (14:1 n-5, 15:1, 16:1 n-9, 16:1 n-7, 16:1 n-5, 17:1, 18:1 n-9, 18:1 n-7, 20:1 n-9, 22:1 n-9, and 24:1 n-9); polyunsaturated fatty acids—PUFA (16:2 n-7, 16:2 n-4, 16:3 n-4, 16:3 n-3, 16:4 n-3, 18:2 n-6, 18:3 n-6, 18:3 n-4, 18:3 n-3, 18:4 n-3, 18:4 n-1, 20:2 n-6, 20:3 n-6, 20:4 n-6, 20:3 n-3, 20:4 n-3, 20:5 n-3, 21:5 n-3, 22:2 n-6, 22:3 n-6, 22:4 n-6, 22:5 n-6, 22:5 n-3, and 22:6 n-3); n-3 PUFA (16:3 n-3, 16:4 n-3, 18:3 n-3, 18:4 n-3, 20:3 n-3, 20:4 n-3, 20:5 n-3, 21:5 n-3, 22:5 n-3, and 22:6 n-3); and n-6 PUFA (18:2 n-6, 18:3 n-6, 20:2 n-6, 20:3 n-6, 20:4 n-6, 22:2 n-6, 22:3 n-6, 22:4 n-6, and 22:5 n-6).

### 2.4. Statistical Analyses

Statistical analyses were carried out using Past4 software 4.12 [24], and graphs were obtained via GraphPad Prism 6 and Microsoft Excel for Microsoft 365 MSO (Version 2310 Build 16.0.16924.20054) 64-bit. The normality and homogeneity of variances were checked using Shapiro–Wilk and Levene tests, respectively. Data transformations were carried out (using ranks) whenever necessary to meet the assumptions. Two-way ANOVA was used to evaluate the influence of sex and reproductive stages (December, March, July and November) on individual fatty acid and fatty acid class (SFA, MUFA, PUFA, n-3 PUFA, n-6 PUFA, n-3/n-6, and docosahexaenoic acid/eicosapentaenoic acid ratio—DHA/EPA) variance. The post hoc Tukey test followed ANOVA. Significant differences were considered when *p* < 0.05.

For principal component analysis (PCA), the variance–covariance matrix was used for a selection of the most representative fatty acids (16:0, 18:0, 18:1 n-9, 20:4 n-6, 20:5 n-3, and 22:6 n-3) and classes of fatty acids (SFA, MUFA, PUFA, n-3 PUFA, and n-6 PUFA) in the liver and plasma samples of each sex. The number of principal components was established by selecting those with eigenvalues greater than 1.

## 3. Results

### 3.1. Male and Female Brown Trout Fatty Acid Analyses

**Figure 1:**
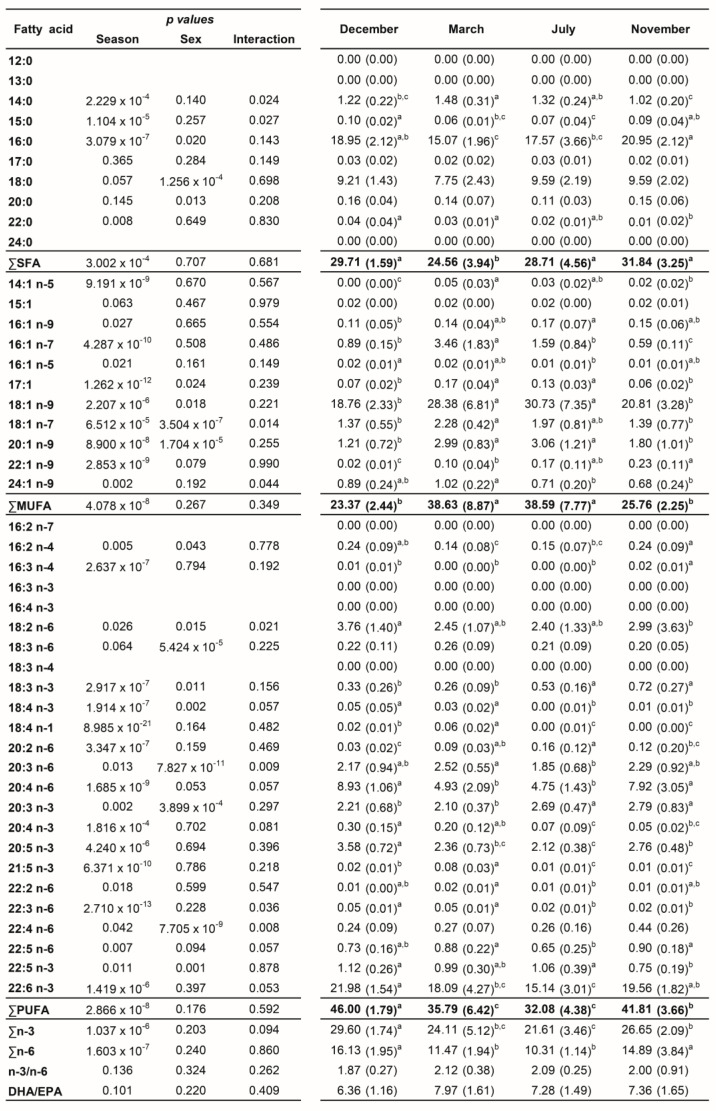
Two-way ANOVA (*p* values of main factors and interaction) and post hoc Tukey test comparisons of the liver fatty acid profiles of female and male brown trout (combined) between the four reproductive stages: spawning capable (December), regressing (March), regenerating (July), and developing (November). Data are shown as mean (standard deviation); n = 12 animals/season (n = 6 males and n = 6 females). Different lower-case letters indicate significant post hoc differences between reproductive stages (*p* < 0.05).

**Figure 2:**
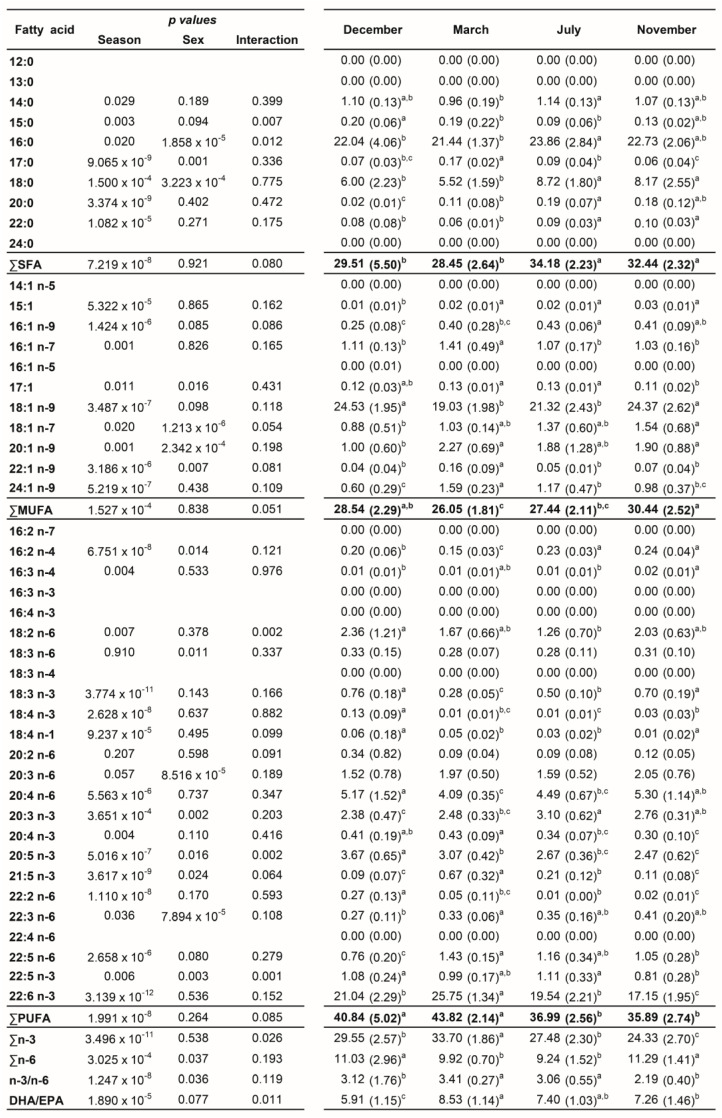
Two-way ANOVA (*p* values of main factors and interaction) and post hoc Tukey test comparisons of the plasma fatty acid profiles of female and male brown trout (combined) between the four reproductive stages: spawning capable (December), regressing (March), regenerating (July), and developing (November). Data are shown as mean (standard deviation); n = 12 animals/season (n = 6 males and n = 6 females). Different lower-case letters indicate significant post hoc differences between reproductive stages (*p* < 0.05).

A total of 45 fatty acids were quantified in both liver (Figure 1) and plasma (Figure 2) samples from brown trout males and females. SFA, MUFA, and PUFA corresponded to 10, 11, and 24 fatty acids, respectively. Of the PUFAs, 10 were n-3 PUFA and 9 were n-6 PUFA. The most representative fatty acids in both types of samples were 16:0, 18:0, 18:1 n-9, 20:4 n-6, 20:5 n-3, and 22:6 n-3, which can be observed by their high percentages of total fatty acids (Figure 1 and Figure 2; Tables S1 and S2).

### 3.2. Liver Fatty Acid Analyses—Sex and Reproductive Stage Differences

Neither sex differences nor interactions between sex and season were found for the distinct fatty acid classes (Figure 1). By analyzing each fatty acid, several differences between sexes and sex versus seasonal interactions were noticed (Figure 1). In contrast, many differences were detected for all classes and most individual fatty acids by comparing the distinct reproductive stages (Figure 1).

**Figure 3:**
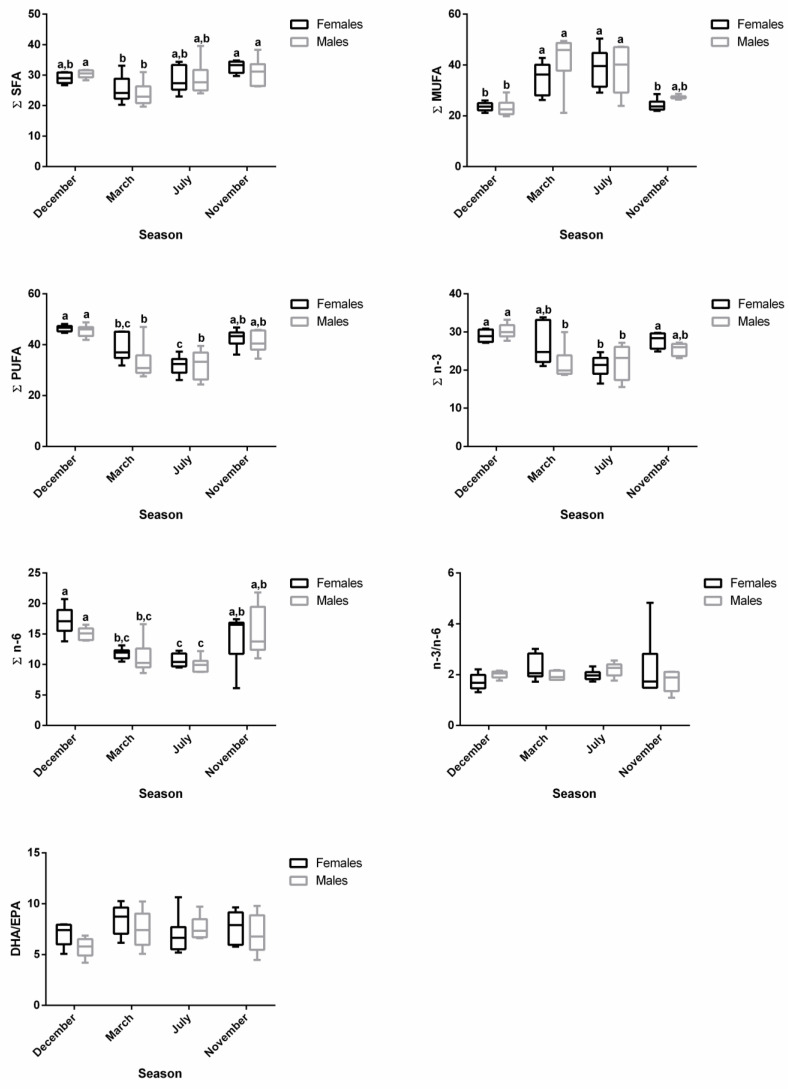
Changes in ∑SFA, ∑MUFA, ∑PUFA, ∑n-3, ∑n-6, n-3/n-6, and DHA/EPA in female and male brown trout liver during four reproductive stages: spawning capable (December), regressing (March), regenerating (July), and developing (November). Data are shown as a median and a minimum-to-maximum range; n = 6 animals/season/sex. Different lower-case letters indicate significant post hoc differences (*p* < 0.05) between reproductive stages within each sex using the Tukey test. The sum (∑) values correspond to the mean of the ∑ of various animals for each class of fatty acids.

For both males and females, ∑SFA was significantly lower in March—regressing stage (females—25.34%; males—23.78%)—compared to November—developing stage (females—32.76%; males—30.93%)—which showed similar levels as the ones found in December—spawning capable—and July—regenerating (Table S1 and Figure 3). For females, ∑MUFA significantly increased in March—regressing stage (34.90%)—and July—regenerating (38.97%)—compared to December—spawning capable (23.60%)—and November—developing stage (24.20%) (Table S1 and Figure 3). With respect to males, the ∑MUFA in March—regressing stage (42.35%)—and July—regenerating (38.22%)—was significantly higher than in December—spawning capable (23.14%) (Table S1 and Figure 3). An inverse pattern was noticed for ∑PUFA in both sexes. ∑PUFA was significantly lower in March—regressing stage (females—38.65%; males—32.92%)—and July—regenerating (females—31.92% and males—32.23%)—than in December—spawning capable (females—46.48%; males—45.52%). The PUFA profile was almost similar for ∑n-3 PUFA and ∑n-6 PUFA in both sexes. n-3/n-6 and DHA/EPA were not influenced by the sex or the reproductive stage of fish, and they varied from 1.72 to 2.27 and 5.70 to 8.44, respectively.

**Figure 4:**
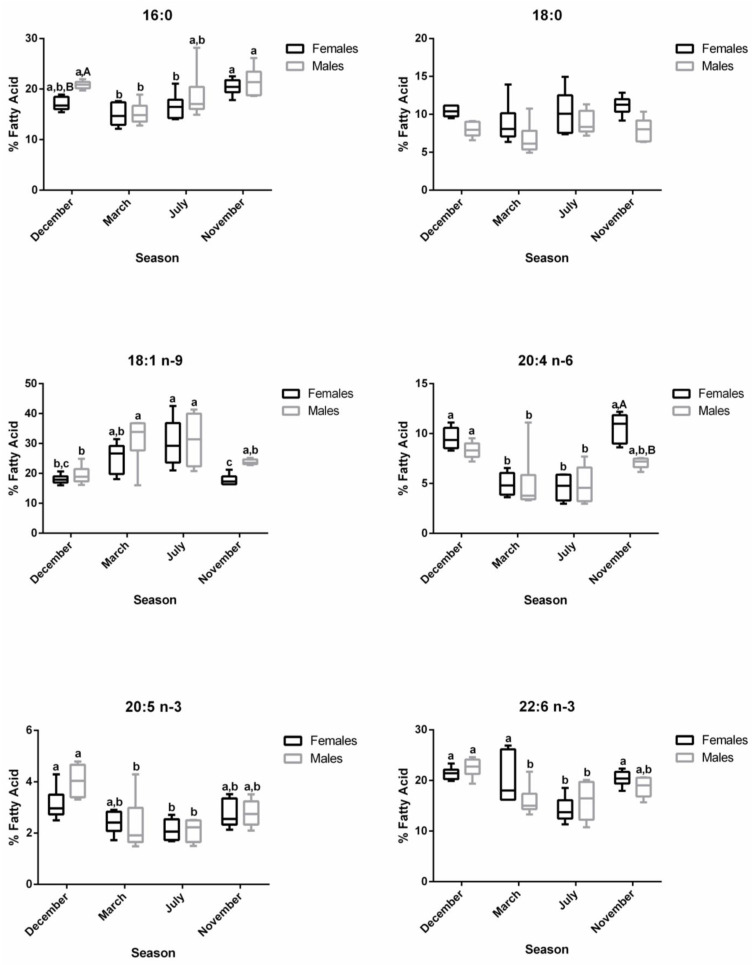
Changes in the percentage of the most representative fatty acids in female and male brown trout liver during four reproductive stages: spawning capable (December), regressing (March), regenerating (July), and developing (November). Data are shown as a median and *a* minimum-to-maximum range; n = 6 animals/season/sex. Different lower-case letters indicate significant post hoc differences (*p <* 0.05) between reproductive stages within each sex using the Tukey test. Significant variations between sexes within a reproductive stage are indicated by different upper-case letters (*p* < 0.05).

The changes along the reproductive stages for the most representative fatty acids of each class are detailed in Figure 4. With respect to SFA, no significant alterations were found for 18:0 between stages. Regarding 16:0, females showed significantly higher levels in November—developing stage—compared to March—regressing stage—and July—regenerating. For males, lower percentages of 16:0 were noticed in March—regressing stage—differing significantly from December—spawning capable—and November—developing stage. For this fatty acid, sex differences were observed in December. From the MUFA, the 18:1 n-9 was the one that stood out the most. In females, the percentage of 18:1 n-9 was significantly higher in July—regenerating—than in December—spawning capable—and November—developing stage; this profile was quite similar in males. By analyzing the three most relevant PUFAs for both males and females, the percentages of 20:4 n-6, 20:5 n-3, and 22:6 n-3 do not vary significantly between December—spawning capable—and November—developing stages. These levels were higher than in July—regenerating—and March—regressing stage—although the differences are not always significant. For 20:4 n-6, a significant difference was found between males and females in November—developing stage.

### 3.3. Plasma Fatty Acid Analyses—Sex and Reproductive Stage Differences

**Figure 5:**
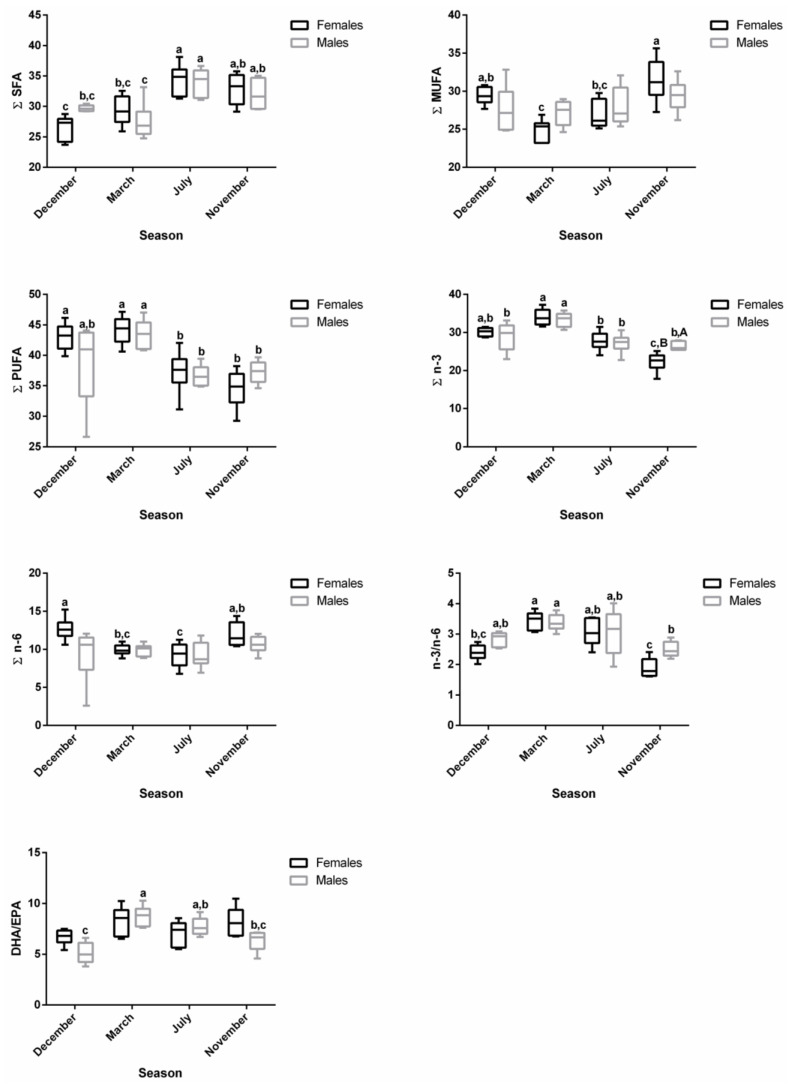
Changes in ∑SFA, ∑MUFA, ∑PUFA, ∑n-3, ∑n-6, n-3/n-6, and DHA/EPA in female and male brown trout plasma during four reproductive stages: spawning capable (December), regressing (March), regenerating (July), and developing (November). Data are shown as a median and a minimum-to-maximum range; n = 6 animals/season/sex. Different lower-case letters indicate significant post hoc differences (*p* < 0.05) between reproductive stages within each sex using the Tukey test. Significant variations between sexes within a reproductive stage are indicated by different upper-case letters (*p* < 0.05). The sum (∑) values correspond to the mean of the ∑ of various animals for each class of fatty acids.

In the plasma samples, the main changes in fatty acid contents were related to reproductive status rather than sex (Figure 2). In females, the ∑SFA profile was significantly lower in December—spawning capable (26.52%)—compared to July—regenerating (34.37%)—and November—developing stage (32.88%)—while in males, significantly lower values were observed in March—regressing stage (27.54%)—compared to July—regenerating (33.99%)—and November—developing stage (32.00%) (Table S2 and Figure 5). The ∑MUFA was not significantly altered in males along the distinct stages, but for females, significantly higher levels were obtained in November—developing stage (31.47%)—than in March—regressing stage (24.92%)—and July—regenerating (26.92%). The ∑PUFA in both sexes were high in March—regressing stage (females—44.16%; males—43.49%)—similarly to December—spawning capable (females—43.05%; males—38.63%)—but differed significantly from July—regenerating (females—37.32%; males—36.66%)—and November—developing stage (females—34.51%; males—37.27%). While the reproductive status did not influence ∑n-6 in males, ∑n-3 was significantly higher in March—regressing stage compared to the other phases. In females, the ∑n-3 was significantly higher in March—regressing stage—compared to July—regenerating—and November—developing stages; ∑n-6 was significantly higher in December—spawning capable—than in March—regressing stage—and July—regenerating. The ∑n-3 in November—developing stage—differed significantly between males and females. Some changes were also reported for the n-3/n-6 and DHA/EPA ratios.

**Figure 6:**
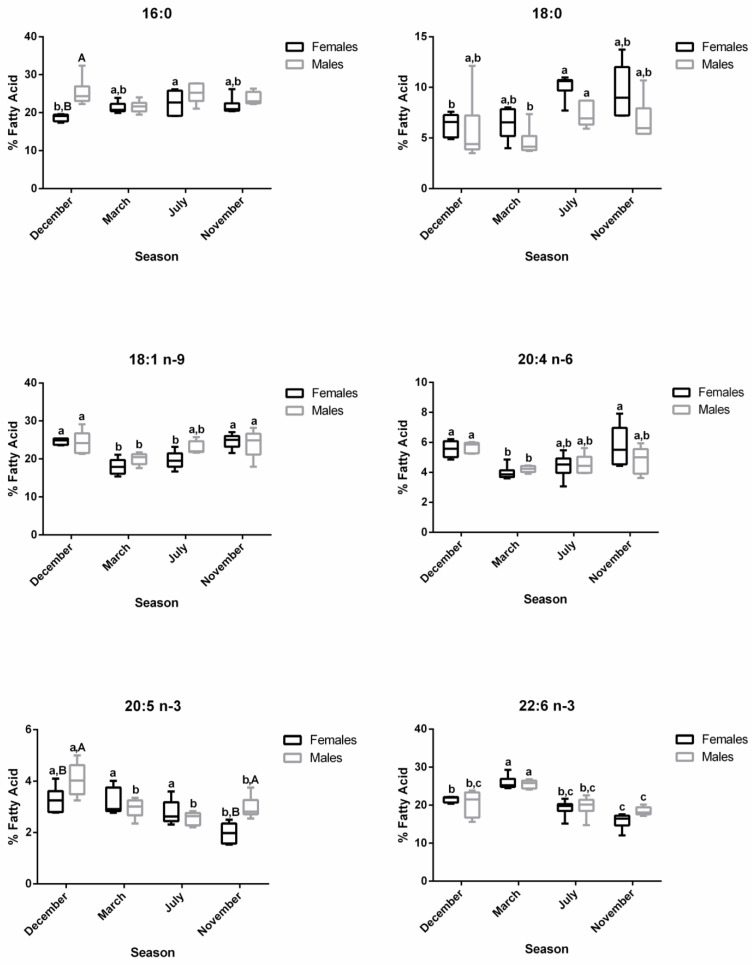
Changes in the percentage of the most representative fatty acids in female and male brown trout plasma during four reproductive stages: spawning capable (December), regressing (March), regenerating (July), and developing (November). Data are shown as a median and a minimum-to-maximum range; n = 6 animals/season/sex. Different lower-case letters indicate significant post hoc differences (*p* < 0.05) between reproductive stages within each sex using the Tukey test. Significant variations between sexes within a reproductive stage are indicated by different upper-case letters (*p* < 0.05).

The profiles of 16:0, 18:0, 18:1 n-9, 20:4 n-6, 20:5 n-3, and 22:6 n-3 in plasma are shown in Figure 6. For 16:0, no changes were observed along the reproductive stages in males, but in December—spawning capable—a sex difference was noticed. For females, the 16:0 and 18:0 levels increased significantly in July—regenerating—versus December—spawning capable. For males, the percentage of 18:0 was the highest in July– regenerating—and differed significantly from March—regressing stage. For females, the 18:1 n-9 showed significantly lower percentages in March—regressing stage—and July—regenerating—versus the remaining stages, while for males, significant differences were observed between March—regressing stage—and December—spawning capable—and November—developing stage. A similar profile was observed for the n-6 PUFA, 20:4 n-6. Regarding 20:5 n-3, in females, the levels decrease significantly in November—developing stage—than in other stages. In males, the percentage of 20:5 n-3 was significantly higher in December—spawning capable—in comparison with the remaining stages. Significant sex differences were obtained for this fatty acid in December—spawning capable—and November—developing stage. For 22:6 n-3, both males and females showed significantly higher percentages in March—regressing stage—than in other stages, and the lowest levels were found in November—developing stage.

### 3.4. PCA Analyses

**Figure 7:**
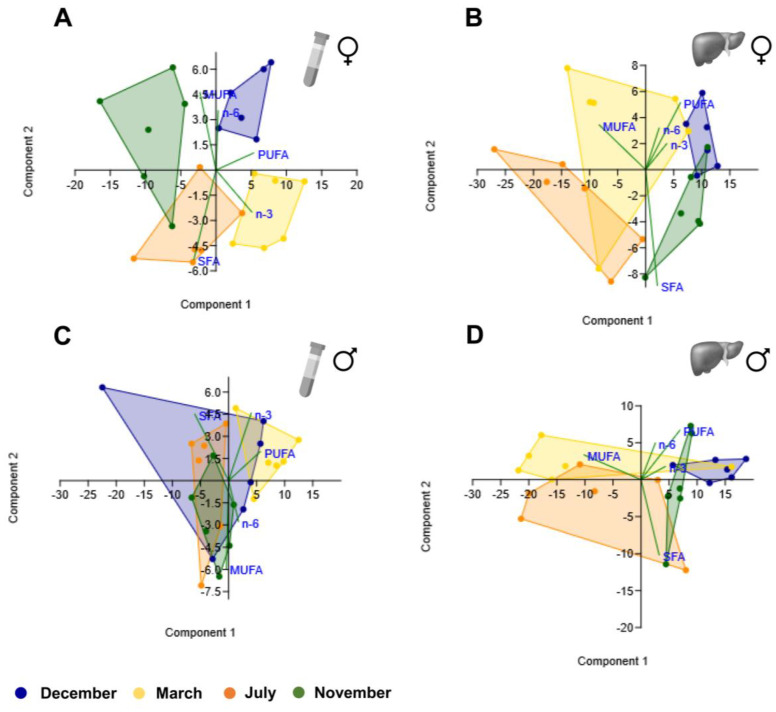
Principal component analysis scatter plot for different classes of fatty acids (SFA, MUFA, PUFA, n-3 PUFA, and n-6 PUFA) in female plasma (**A**) and liver (**B**) brown trout samples and male plasma (**C**) and liver (**D**) samples.

**Figure 8:**
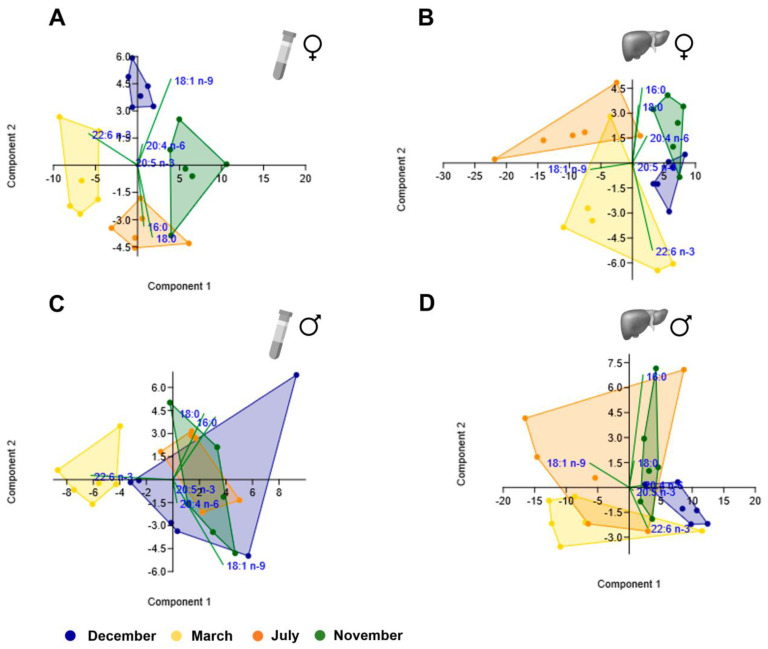
Principal component analysis scatter plot for the representative fatty acids (16:0, 18:0, 18:1 n-9, 20:4 n-6, 20:5 n-3, and 22:6 n-3) in female plasma (**A**) and liver (**B**) brown trout samples and male plasma (**C**) and liver (**D**) samples.

From the different scatterplots shown in Figure 7 and Figure 8, it is evident that the ones obtained from female plasma samples (Figure 7A and Figure 8A) discriminate the different clusters corresponding to the four reproductive stages better, which did not occur so clearly for female liver samples (Figure 7B and Figure 8B) nor male plasma (Figure 7C and Figure 8C) and liver samples (Figure 7D and Figure 8D). The clusters were distinct when comparing plasma and liver scatterplots within each sex (Figure 7A–D and Figure 8A–D), but they seem to overlap substantially when comparing liver profiles between sexes (Figure 7B,D and Figure 8B,D).

For PCA analyses in the female plasma of fatty acid classes, a variance of 99.9% was accounted for by the first three components (PC1—75.9%; PC2—21.7%; PC3—2.3%). The higher PC1-positive loadings were found with respect to PUFA and n-3 PUFA. In male plasma, the first three components explain 99.9% of the variance (PC1—77.5%; PC2—19.5%; PC3—2.9%), and the highest PC1-positive and -negative loadings were for PUFA and SFA, respectively. In the liver, both sexes had more than 80% of variance explained by PC1, which showed the highest positive and negative loadings with respect to PUFA and MUFA, respectively.

In the plasma PCA analysis of the most representative fatty acids, for females, the highest positive loading with PC1 (which explained 60.2% of the variance) was with 18:1 n-9 belonging to MUFA, while the loading was strongly negative for 22:6 n-3 (n-3 PUFA). The same pattern was observed in the male plasma. In the liver, the opposite happened for females and males. In both sexes, the highest negative loading with PC1 (which explained more than 80% of the variance) was found for 18:1 n-9.

## 4. Discussion

### 4.1. FA Profiles in Cultured Brown Trout

Brown trout at the selected reproductive phases were characterized in detail in our previous study in terms of fish biometry; blood; plasma or serum biochemical parameters; some sex-steroid and gonadotropin hormonal levels; and gonad morphology [18]. The current study explores different concepts and targets, which nonetheless expand and complement the morphofunctional description of this species at different phases of the reproductive cycle. The study evaluated the influence of different reproductive stages and sex as factors that can interfere with the fatty acid composition in the liver and plasma of cultured adult brown trout individuals. In general, herein, the gonad maturation status influenced the liver and plasma FA profiles more than sex, although sex differences for specific FAs have been detected. In a previous study by others, using cultured brown trout, a seasonal variation of MUFA and PUFAs in muscle was not observed, contrary to what was observed in wild specimens [2]. In the present study, liver and plasma samples suggested that the profile of the used trout behaved similarly to wild-type specimens.

Here, in both liver and plasma, irrespective of the reproductive stage or sex, the most representative FAs were 16:0, 18:0, 18:1 n-9, 20:4 n-6, 20:5 n-3, and 22:6 n-3. This evidence indicates that the variations detected among these FAs could be used as relevant targets for monitoring the reproductive status of brown trout. Interestingly, some of these FAs (16:0, 18:1 n-9c, and 22:6 n-3) were the predominant ones in rainbow trout eggs [25], reiterating their broad importance in the reproductive process. In the case of 22:6 n-3, its relevance has been linked, for instance, with the normal embryonic development in trout [26]. Further, in this study, the higher levels of 20:4 n-6 and 20:5 n-3 in the liver and plasma of mature fish (December—spawning capable) compared to March—regressing—and July—regenerating stage—suggest the crucial role of these two fatty acids in the gonad maturation process in both sexes. Examples from different fish support the influence of 20:4 n-6 or its metabolites with respect to oocyte maturation [27,28]. Previously, in macrostigma trout liver, 16:0 was the most abundant SFA in both sexes, with percentages within the range of values found in the present study [10]. Further, in mature Black Sea trout (*Salmo trutta labrax*), 16:0 was the most representative FA of the class not only in the liver but also in other tissues [29]. The 18:0 SFA was the second most quantified SFA in the liver of brown trout, as reported in other fish studies [10,14,16]. Also, as observed here, several studies recognize 18:1 n-9 as the predominant MUFA in different tissues of trout species [10,29,30]. In the present study, the major n-6 PUFA was 20:4 n-6, while 22:6 n-3 and 20:5 n-3 were the most representative n-3 PUFA. In line with our data, high levels of 20:4 n-6 and 22:6 n-3 have been detected in the liver of distinct freshwater fish species [14,15,31].

### 4.2. Liver FA Class Changes—Sex and Reproductive Stages

In this study, males and females had substantial overlapping fatty acid profiles in the liver. For instance, a similar pattern was obtained in the n-3 PUFA levels in both sexes, but differences between males and females have already been reported in macrostigma trout liver [10]. In both sexes, higher MUFA levels and lower PUFA contents (either n-3 PUFA or n-6 PUFA) were noted during the regressing and regenerating stages (March and July, respectively). An inverse pattern was observed along the spawning capable (December) and developing stages (November). The changes in ∑MUFA were mainly due to 18:1 n-9. For n-3 PUFA and n-6 PUFA, 22:6 n-3, 20:5 n-3, and 20:4 n-6 were the most representative fatty acids. In agreement with our results, in the liver of Pacific herrings (*Clupea harengus pallasi*), it was observed that 18:1 n-9 levels decreased significantly in spawning versus non-spawning fish, while 22:6 n-3 and 20:4 n-6 were higher in spawning fish [32]. In the lesser amberjack (*Seriola fasciata*), the liver of pre-spawning fish also had increased levels of total MUFA, which decreased at the spawning stage [33]. This relationship can be explained by the fact that as the spawning phase approaches, the need for energy metabolization may lower 18:1 n-9 levels. Further, the ∑MUFA was higher during the warmer months, corresponding to the regressing and regenerating stages (March and July), which may be related to the high feeding rate, as is suggested to occur in *Salmo trutta* [34]. This fact was previously suggested because the highest percentages of the MUFA of neutral lipids in three *Salmo trutta* sub-species (in muscle) were found during the spring season [34]. On the contrary, wild brown trout muscle showed higher PUFA levels during the autumn and winter seasons and exhibited lower contents in spring and summer [2]. The present study also found these PUFA seasonal changes in liver samples. In the muscle of different freshwater fish, evidence points to higher values of PUFAs, namely n-3 PUFA, and they are associated with lower temperatures [35], as has been observed in November and December. In three subspecies of *Salmo trutta*, the PUFA contents of neutral lipids in muscle were also increased in cold seasons, including the spawning period [34]. The higher PUFA contents in brown trout during the colder seasons reiterate the association of increased unsaturated FA biosyntheses in adjusting membrane fluidity [36,37].

In the present study, the SFA levels were low in both sexes during the regressing stage (March), corresponding to the beginning of the spring season. The same profile was reported for neutral lipids in Black Sea trout [34].

### 4.3. Plasma FA Class Changes—Sex and Reproductive Stages

Seasonal variations in the ∑SFA were more evident in plasma than in the liver despite the similar pattern. Lower SFAs were observed during the spawning (December) and regressing (March) stages, as previously described in the muscle of wild fish from the same species [2]. The discriminatory variations between stages in male and female plasma were mostly related to ∑SFA and ∑PUFA and ∑PUFA, namely ∑n-3 PUFA, respectively. This finding suggests that PUFAs are crucial for reproduction in both sexes, namely for gonad maturation and spawning events. In accordance, evidence from female fish treated with PUFA diets showed increased gonadal maturation [8] and spawning capacities than controls [38]. In male fish, PUFA supplementation caused an increase in sperm count and spermatocrit [38]. Further, sperm motility was negatively affected in the fish exposed to a reduced n-3 PUFA diet compared to the individuals taking the control diet [39].

From spawning (December) to regressing (March) stages, the n-3/n-6 ratio increased in the plasma samples of both sexes, coinciding with the winter season. This profile was similar to the one described in the muscle from *Salmo trutta macrostigma* [34] and three tilapia species [40]. The relation between the increase in n-3/n-6 and lower gonadosomatic index (GSI) values was previously reported [34], and it is in line with our data since it was observed that brown trout from the same geographical location had low GSI at the regressing stage (March) [18].

## 5. Conclusions

This study reinforces the observation that fatty acid compositions are distinct at each reproductive stage both in the liver and plasma of brown trout adults when using the same commercial diet. Nevertheless, it must be stressed that a diet with a distinct fatty acid profile may lead to differences in relation to the data reported here. After, analyzing the same animals, it was concluded that the blood biochemical parameters of cholesterol and total protein better distinguished gonadal stages in both sexes, while the sex hormones 11-ketotestosterone and testosterone in males and 17β-estradiol in females were quite explicative of reproductive stage variations [18]. Here, the liver and plasma fatty acid patterns were unique for each sex. There were significant sex differences in 35.6% and 37.8% of individual fatty acids in liver and plasma samples, respectively. Overall, the most representative fatty acids from each class were 16:0, 18:0, 18:1 n-9, 20:4 n-6, 20:5 n-3, and 22:6 n-3. It became evident that plasma from females (and less clearly liver) could discriminate clusters for each reproductive stage based on the most representative fatty acids. In this way, these fatty acids can be investigated in the future as biomarkers of the maturation state of female gonads throughout the reproductive cycle, at least in aquaculture and probably in the wild. Eventually, the plasma fatty acid profile may be even more discriminative and promising for predicting ovulation times in aquacultures. However, to be considered a useful tool, sampling throughout the reproductive cycle would have to be tighter than the one used in this study in terms of the time window. New data can also be used as a baseline reference for interpreting fatty acid metabolic disturbances caused by multiple factors.

## Data Availability

Data are contained within the article and Supplementary Materials.

## Supplementary Materials

The following supporting information can be downloaded at https://www.mdpi.com/article/10.3390/biology12111434/s1. Table S1: Fatty acid composition (percentages of total fatty acids) of female and male brown trout liver in the four reproductive stages: spawning capable (December), regressing (March), regenerating (July), and developing (November). Values correspond to the mean (standard deviation). The sum (∑) values correspond to the mean of the ∑ of the various animals for each class of fatty acids. Table S2: Fatty acid composition (percentages of total fatty acids) of female and male brown trout plasma in the four reproductive stages: spawning capable (December), regressing (March), regenerating (July), and developing (November). Values correspond to the mean (standard deviation). The sum (∑) values correspond to the mean of the ∑ of various animals for each class of fatty acids.
